# Piloting, testing and scaling parental training: a multi-partnership approach in Côte d’Ivoire

**DOI:** 10.3389/fpubh.2023.1106565

**Published:** 2023-08-15

**Authors:** Romuald Anago, Tiphaine Forzy, Sosthene Guei, Charlotte Pelras, Samuel Ramde, Camille Tevenart, Julieta Vera Rueda, Karen Macours

**Affiliations:** ^1^Innovation for Poverty Action, Abidjan, Côte d'Ivoire; ^2^Independent Consultant, Abidjan, Côte d'Ivoire; ^3^Transformer L’éducation Dans Les Communautés de Cacao, Abidjan, Côte d'Ivoire; ^4^Paris School of Economics, Paris, France; ^5^Institut National de Recherche Pour L’agriculture, L’alimentation et L’environnement (INRAE), Paris, France

**Keywords:** early childhood development, MEL, scaling, parental training, randomized controlled trial

## Abstract

**Background and objectives:**

Early Childhood Development is high on the policy agenda in Côte d’Ivoire, where the government has identified it as part of its overall approach to improve human capital outcomes. This paper describes a multi-partner approach to piloting, monitoring, adaption, testing and scaling of parental training for ECD. It discusses the learnings from the pilots, and present early evaluation results from two RCTs, focusing on parental participation in trainings and acceptability of messages, with the objective to inform national scaling strategies. As such, this paper illustrates how “MEL systems contributed to ensuring that positive early childhood development (ECD) outcomes were improved as interventions were seeking to achieve scale,” one of the research questions outlined in the call description for the special issue. The paper further provides a real-world example of “How MEL systems can support contributions and buy-in from a variety of stakeholders as ECD interventions (seek to) achieve impacts at scale (e.g., through the public system)?

**Methods:**

Five training approaches to improve caregivers’ knowledge and practices around nutrition, preventive health, stimulation, and disciplining were piloted at small scale between 2018 and 2020. An intensive process evaluation was embedded to identify strengths and weaknesses, adapt through an iterative phase, and ultimately make recommendations for their scale up against 11 defined criteria. In early 2021, the two most promising approaches were scaled through two clustered randomized control trials to more than 150 villages each. A cost-effectiveness study was designed in consultation with government stakeholders, centered around targeting different caregivers and decision makers in the household and the extended family and on enhancing community interactions around ECD.

**Results:**

The evaluation of the five pilots identified one model recommended to be scaled, and one other model to scale after further adaptations. Monitoring and evaluation data from the two models at scale show high levels of participation and acceptability of core messages. Experimental variations involving community champions and fathers increase participation.

**Conclusion:**

The iterative and multi-partner process led to two models of parenting training that have wide acceptability. Future work will analyze impacts on cognitive and socio-emotional outcomes, together with cost analysis.

## Introduction

The government of Côte d’Ivoire has made early childhood development (ECD) a strategic policy priority with the goal to improve the country’s human capital indicators, stimulate economic growth and alleviate poverty. This commitment is reflected in the Multisectoral Government Project for Nutrition and Early Childhood Development, a multi-stakeholder investment of 60.4 million USD and the creation of the National Council for Nutrition, Food and Early Childhood Development. The policy focus is motivated by Côte d’Ivoire’s strong lags in human capital development. Despite being a middle-income country, and some improvements in human capital over the last 10 years, Côte d’Ivoire still ranks only 158 out of a total of 171 countries in the World Bank’s Human Capital Index in 2020, a composite measure of health and education. The ranking reflects a low probability of survival until age 5, low schooling and low learning, which are all at levels below the average for Sub Sahara Africa, and much below the average for lower middle-income countries (1). Moreover, evidence of low human capital investments is apparent at very early ages [with adequate meal frequency among children 0–23 months only at 48 percent, and pre-primary enrollment at 10% (1)], a particular concern given the importance of the early years for a child’s brain development (2), and motivating the government’s prioritization of early childhood development.

Learning from international evidence on the powerful potential of early childhood stimulation (3–5), parenting training focused on nurturing, playful and loving care for children in the first years of life is an important component of the national strategy. Yet little empirical evidence existed on the effectiveness of parenting training approaches in the Ivorian context, and even less on optimal ways to embed such training into existing government structures. Linking early childhood development programs to existing social welfare systems with established administrative capacity and local community networks has been identified as a promising way to scale (2) and the international evidence on such approaches is growing (6–9). There are however many open questions on how to sustainably and effectively scale parenting interventions in Côte d’Ivoire, and more widely in low- and middle-income countries (10–13). Part of these questions are operational (such as how to leverage existing infrastructure and staff while assuring the necessary quality of content and delivery of training), while others relate to understanding the decision-making process among caregivers and their communities when deciding to adopt new parenting practices and behaviors (14, 15).

**Figure 1:**
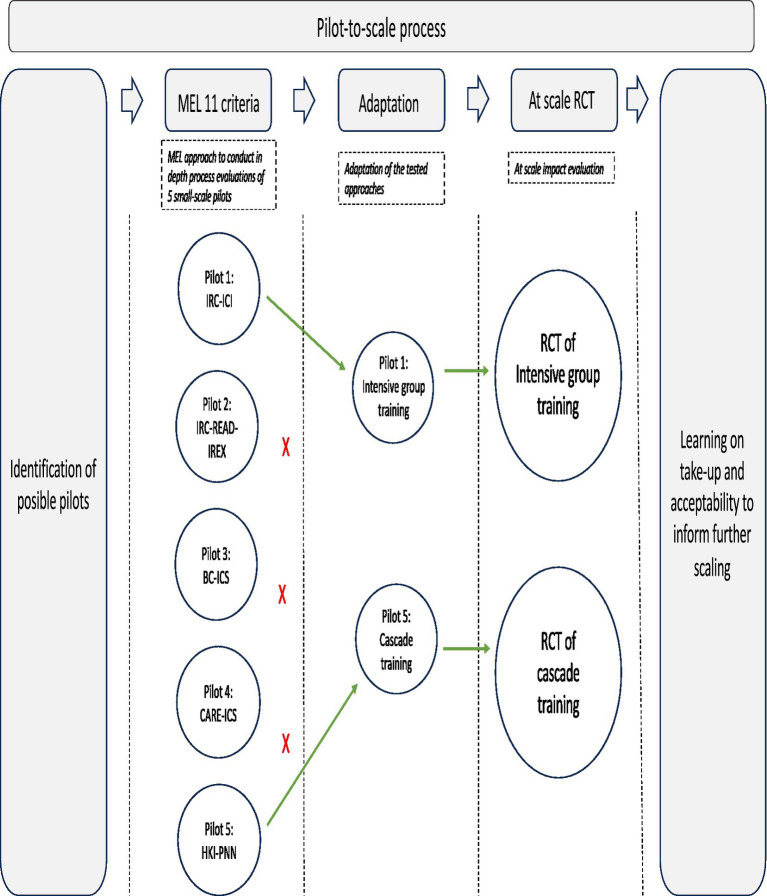
Road-map diagram MEL in pilot-to-scale approach.

This paper documents the development, implementation, and initial findings of a pilot-to-scale program in Côte d’Ivoire, aimed at tackling these operational and conceptual challenges, and highlights the essential role of monitoring, evaluation and learning in the pilot-to-scale process. The pilot-to-scale process was led by TRECC, which stands for “Transformer l’Éducation dans les Communautés de Cacao” or (in English) “Transforming Education in Cocoa Communities program,” an initiative funded by the Jacobs Foundation, the Bernard van Leer Foundation and UBS Optimus Foundation, with the aim to foster close collaboration between public and private institutions to help ensure that children in Côte d’Ivoire are afforded a good start in life and quality education. TRECCs approach is explained in the next section. TRECCs pilot-to-scale process built in a strong MEL (Monitoring, Evaluation and Learning) approach to maximize learnings from pilots and make evidence-based scaling decisions illustrated in Figure 1. The paper first outlines the MEL approach used to conduct in-depth process evaluations of five different small-scale pilots and explains how this led to an identification of the two most promising candidate models for scaling. The pilots further revealed that many of the tested approaches ultimately relied on components that were deemed hard to sustain after the end of the pilots and that there was overall limited vision on cost management when scaling. The next section explains how subsequently, the two pilot approaches deemed most likely to overcome those challenges were rolled out at a larger scale, with a randomized quantitative impact evaluation built in, to obtain estimates of cost-effectiveness at scale, and rigorously test different design options within them. The implementation at scale showed some concerns with low participation and knowledge transmission in the cascade model approach relying on volunteer trainers, but also some encouraging results on design features that could help offset participation constraints. As such, this paper contributes to the growing literature specifically discussing the contributions of MEL approaches to the global ECD agenda (16, 17) and relates to other recent work documenting the importance of MEL for specific ECD interventions (18, 19).

## TRECC approach to scaling

To inform scaling of early childhood parenting training by the Ivorian Government, TRECC, supported the piloting, testing, adaptation, and scale up of various multi-partner implementation models. TRECC mobilized a range of stakeholders including the Government of Côte d’Ivoire and pooled resources and expertise toward the common goal of improving ECD outcomes through a public-private partnership approach, catalyzing an initial investment of approximately 10 million CHF (see Annex A for details on TRECC’s approach and a list of partners). The subject matter technical expertise for caregiver training content and approaches developed and adapted by international NGOs and think tanks, was brought together with the mandate, long-term interests, and infrastructure of various line ministries, resources from the private sector and philanthropic foundations, and M&E (monitoring and evaluation), impact evaluation, and ECD expertise from local research organizations and global academia. To assure effective allocation of resources and provide lessons for contextualized actionable solutions for scaling, TRECC built in a deliberate process of evidence generation and data-driven decision-making.

In a first step, several promising approaches were selected for piloting. The purpose of the pilot phase was to adapt and test approaches with proven effectiveness in other settings in the context of rural Côte d’Ivoire and assess their potential for integration in national programs. To assure optimal learning from this pilot phase, a robust and collaborative Monitoring, Evaluation and Learning (MEL) approach was designed to accompany the pilots. Apart from informing the government’s policy, learning from MEL was also seen as a key input into decision making regarding the funding of any scaling initiatives by the industry partners who were providing the operational funding for the pilots.

After iterative adaptations based on the results of the pilot phase, the most promising approaches were selected to be rigorously tested at larger scale. A specific objective of this testing-at-scale was to seek government buy-in for integrating the most effective approaches into government plans for national scaling.

## Learning from small-scale pilots

Drawing on the strengths of the different partners, five approaches to improve caregivers’ knowledge and practices and promoting nurturing care for healthy child development outcomes were piloted at small scale between 2018 and 2020 in cocoa growing areas. The approaches to be piloted were identified through TRECCs broad network of partners. Selection was based on demonstrated effectiveness in other contexts (20–22), presence of NGO partners in the country to provide technical support, and interests from the government partners, in light of the ultimate goal of scaling as part of government policies. The approaches were adapted to Côte d’Ivoire, and then tested on a small number of communities along with monitoring and process evaluation activities focused on collecting empirical evidence on the early steps in the theory of change of parenting training programs. The goal was not primarily to compare the models, but rather to document for each of them whether basic operational and acceptability criteria for successful scaling were satisfied.

At the pilot stage, NGOs implemented the programs either directly or indirectly–by training the government local services and strengthening local capacities. Table 1 describes the partners, activities, targets, curriculums, frequency of the training, trainees, and costs of the pilots (see Annex B1 for details). Different implementation modalities and partnerships were tested, but also different approaches to beneficiary targeting, selection of trainers, and intensity of training. All curricula incorporated common aspects of nurturing care along various domains of ECD. In 4 out of 5 pilots, trainings were in group, while the 5th pilot tested a combination of group trainings and home visits. Some pilots incorporated other interventions in addition to parenting training. Pilot 2, for instance, with the highest cost per child, included the creation of community learning centers as well as training in financial literacy and income generation. In contrast, pilot 5 with the lowest cost per child, is uniquely focused on parental ECD training.

**Table 1:** Partners, components, parental training organization and costs of the 5 pilots.

	Partners *technical **government ***financial	Intervention details/activities	Target beneficiary groups	Parental training curriculum	Frequency of the training activities	Profile of trainers	Training-of-Trainers (ToT) and supervision	Average cost per child
1	International Rescue Committee (IRC)* International Cocoa Initiative (ICI)* Ministry of the Family, Women and Children (MFFE)** Touton***	Promote a nurturing environment for children through parental training in the Family Makes the Difference (FMD) curriculum Preschool (CACE) construction, equipment and staffing to implement ECD activities Child Labor Monitoring and Remediation System (CLMRS)	240 parents of Touton cooperatives (max 25 per group) 600 children aged 0–5 75 pre-primary school children aged 2–5	Nurturing care, early learning, developmentally appropriate guidance, adult stress management, positive discipline and communication practices, effects of toxic stress and violence on child brain development, health and nutrition best practices	10 training sessions (2 h per session) scheduled according to beneficiaries and Government partners’ availability over a 5 month period.	Training provided by 12 social workers (7 men & 5 women) who are state agents 2 community members for each village (6 in total) also supported social workers in leading the FMD sessions.	ToT and Supervision and direct observation of FMD sessions by IRC staff	about $302 per child
2	International Rescue Committee (IRC)* Rights Education And Development Center (READ)** The International Research & Exchanges Board (IREX)** Ministry of the Family, Women and Children (MFFE)** Mondelez***	Promote a nurturing environment for children through parental training in the Family Makes the Difference (FMD) curriculum Creation of READ community learning centers to build parenting skills and improve the physical, intellectual, and social–emotional well-being of young children’s Training of VSLA members on financial literacy and income generation with READ and IREX’s support to sustain the basic operating costs of the Community center.	200 parents mainly women members of existing Village Savings and Loan Associations (max 26 per group) (VSLA) groups 800 children aged 0–5 500 community members	Nurturing care, early learning, developmentally appropriate guidance, adult stress management, positive discipline and communication practices, effects of toxic stress and violence on child brain development, health and nutrition best practices.	11 training sessions (2 h per session) scheduled according to communities and government partners’ availability over a 4 month period	Training provided by social workers who are preschool educators, who had received specific training in early childhood development during their initial training at the National Institute of Social Training (INFS)	ToT and supervision and direct observation of FMD sessions by IRC staff	about $320 per child
3	Investing in Children and their Societies (ICS)* BC-SACO*(both implementing and financial partner)	Contextualize and implement parental training in Skillful Parenting curriculum Connect families to social support services Training of VSLA members on financial literacy and income generation/diversification	307 parents (max. 25 per group) 900 children aged 0–8 300 partners of farmers sensitized. Community members and 12 social services providers and government workers sensitized on parenting.	Age-appropriate parenting, functional adult relationships; roles and responsibility of skillful parents; Early Childhood Development; nutrition; self-esteem and self-care; values and discipline; communication; child protection.	Weekly training (2–3 h per session) delivered at the Farmer Field School meeting place during 3–4 months	Training provided by Farmer field school coaches (agronomists, 11 men and 2 women).	BC Coaches trained by ICS staff and receive a facilitation pack for Skillful Parenting curriculum.	About $201 per child
4	Investing in Children and their Societies (ICS)* CARE* Mars***	Parental Training in Skillful Parenting curriculum to VSLA groups. Connecting families to social services Training of VSLA members on financial literacy and income generation/diversification	225 parents of 9 VSLAs (max 30 per group) 1000 children aged 0–18 years old	Family relations; roles and responsibility of a skillful parent; self-esteem and self care; ECD; nutrition; values and discipline; communication; child protection; family budgeting	Weekly training (2 h per session) during 3 months	Training provided by Volunteer VSLA promoters trained as community facilitators & cofacilitators.	Promoters trained by ICS staff and receive a facilitation pack for Skillful Parenting curriculum.	About $61 per child
5	Helen Keller International (HKI)* Program National de Nutrition (PNN), part of the Ministry of Health and Public Hygiene (MSHP)** Blommer*** Hershley***	Training of Master trainers, Health workers and Community Volunteer agents. Group trainings whereby mothers, grandmothers, fathers and any other closest family members are introduced to Care for Child Development (C4CD) curriculum and Essential Nutrition Actions/Essential Hygiene Actions (ENA/EHA) Home visits	4531 caregivers, specifically pregnant women, mothers of children up to age 2, fathers, and grandmothers (max 20 per group)	Nutrition, health and hygiene practices to enhance maternal and child nutrition; early childhood development practices through appropriate play and communication	8 monthly sessions (1–2 h per session) during 8 months Home visits	Training provided by 136 Community volunteers with no specific profile	ToT and Supportive supervision to reinforce knowledge and skills over time provided by HKI and PNN staff once a month for the first few months, then periodically as needed thereafter.	About $47 per child

Table the main information on all interventions in column 2 and provides more details of parental training (the focus of this article) in the remaining columns. Costs include set-up costs of the NGOs that given the small scale of the pilots can be a relatively high share, in particular for pilots with a relatively small number of beneficiaries (first 4 pilots). Costs per beneficiary are likely to be smaller in the scaled-up versions. Costs are calculated using average exchange rates of 2018.

TRECC and its partners purposely embedded an intensive process evaluation into the pilot stage to identify strengths and weaknesses of each pilot. The results from the process evaluation informed iteratively adaptations and ultimately recommendations for scaling. IPA, an NGO with expertise in data collection and management for monitoring and evaluation purposes, provided the technical support to the implementing partners to design and implement their own monitoring systems, including assistance on data collection methods, quality checks and analyzes. Complementing these administrative data collected by the partners themselves, IPA also directly engaged in independent primary data collection through a combination of methods. Beneficiary parents were surveyed for input and their knowledge tested at the start and end of trainings. The number of beneficiary parents interviewed differed for each pilot (see Annex B3), reflecting the different scales of the pilot. For the first pilot, for instance, all 240 beneficiary parents were targeted to be interviewed at baseline (before the start of the pilot), at midline (during program implementation) and at endline (after the program ended). The baseline survey aimed at gathering beneficiaries’ characteristics at the beginning of the pilot, including levels of need. The midline collected data on beneficiaries’ feedback about the implementation of the program and rates of non-attendance. The endline checked beneficiaries’ learning and collected overall feedback from the participants. In addition, non-beneficiaries were also interviewed at baseline to verify the program reached the farmers who needed the intervention the most. Focus groups were organized to complement the quantitative data collection with qualitative insights and spot-checks were conducted to observe the training implementation and check reliability of monitoring data. Finally, key informant interviews provided perspectives of trainers, implicated government officials, community leaders, and partners’ implementing staff. The information collected was used to provide real-time feedback to the different implementing partners and other stakeholders, to allow for course correction, when needed. In addition, the combination of the administrative data and primary data was used to produce a comprehensive report after the end of the pilot for the final assessment.

Each pilot was assessed by IPA on 11 common pre-agreed criteria (specified in Table 2) using these different information sources, which provided measures of relevance, results, costs and operational management, capacity to learn, improve and innovate, and sustainability. The criteria together evaluate the preconditions for pilot approaches to be scalable, considering both vertical, horizontal and organizational scaling (see Annex A2). For any parenting intervention to be able to reach impact at scale, it first needs to assure that trainings with adequate transmission of knowledge can be organized, and parents of children at risk of development delays can be identified, are likely to attend trainings, and accept the trainings’ messages. Moreover, for implementation models to be scalable they need to build on realistic assumptions of available human resources and have cost structures that can be sustained (see (12, 23) for related in-depth discussion of factors to account for when scaling ECD interventions). Empirical evidence on these criteria requires the right type of data, coming from process evaluation and monitoring activities (24, 25). Validating these initial steps in the theory of change was deemed a critical step before moving to testing impact at scale.

**Table 2:** Evaluation outcomes for the 5 pilots on 11 criteria and overall recommendation.

Evaluation criteria	1. IRC-ICI	2. IRC-READ-IREX	3. BC-ICS	4. CARE -ICS	5. HKI
**Relevance**					
Targets an important need in the community	- about 50% of children in the target communities are not developmentally on track (see Methods for definition) - 85% beneficiaries are caregivers of at least one child in the target age group (0–8) - needs are at least as great among beneficiaries as non-beneficiaries	- about 50% of children in the target communities are not developmentally on track (see Methods for definition) - 95% beneficiaries are caregivers of at least one child in the target age group (0–8) - needs are at least as great among beneficiaries as non-beneficiaries	- 48% of beneficiaries are caregivers of at least one child in the target age group (0–8 yrs) - working through cooperatives does not result in targeting the population that is most in need in the communities	- most of parents in the target communities not aware of the appropriate means of child stimulation - 83% beneficiaries are caregivers of at least one child in the target age group (0–8) - needs are at least as great among beneficiaries as non-beneficiaries	- less than half of parents can name 2 stimulation practices (baseline survey) -57% of beneficiaries are caregivers of at least one child in the target age group (0–2 yrs)
Aligns with the priorities of the donors	- a clear will of the donors to scale-up the program	-donors are not willing to scale resources centers as they were implemented	- unclear alignment with BC’s approach and expected results (tackle directly child labor)	- unclear involvement and will of donors to scale-up the program	- a clear will of the donors to scale-up the program
**Results: outputs and direct outcomes**					
Outputs delivered	- Implementation of planned training sessions and government workers’ training completed -85% of beneficiaries attended at least 80% of the trainings	- implementation of the planned training sessions completed - failure to implement community centers and related activities due to low engagement of the communities - 98.5% of beneficiaries participated to at least 8 sessions	- implementation of planned training sessions completed -82% of beneficiaries completed all 9 modules through main sessions or catch-up sessions	- implementation of planned training sessions completed -56% of beneficiaries attended at least 7 out of the 9 training modules	- implementation of planned trainings and home visits completed - about 80% of the beneficiaries attended 8 sessions per month during the last 3 months of the pilot
Achieves direct outcomes (see Supplementary Table SA1 for details)	- significant improvement on 4 out of 6 knowledge and practice indicators	- significant improvement on nutrition and child protection related knowledge - significant improvement on reported practices - no improvement on the indicators related to the community centers	- significant improvement on 3 out of 6 knowledge and practice indicators	- significant improvement in 1 out 2 practice indicators	- significant improvement on nutrition related knowledge - no improvement on hygiene related knowledge and ECD reported practice
Beneficiaries’ feedback about the program is positive	−71% of beneficiaries reported that they would recommend the training sessions	−99% of beneficiaries reported that they would recommend the training sessions	−99% of beneficiaries reported that they would recommend the program	- 93% of beneficiaries reported that they would recommend the program	−90% of beneficiaries reported that they would recommend the program
**Costs and operations management**					
Costs are well managed/cost scale-up vision	- efficient use of pilot’s resources - clear partners’ vision of cost management when scaling	- efficient use of pilot’s resources - incomplete partners’ vision of cost management when scaling	- efficient use of pilot’s resources - no information available on in-kind contributions and opportunity costs	- exceeded initial budget - no information available on in-kind contributions and opportunity costs - incomplete partners’ vision of cost management when scaling	- efficient use of pilot’s resources - incomplete partners’ vision of cost management when scaling
Project management is successful	- project implemented as planned -excellent cooperation between partners	- substantial delays in the implementation of the community centers	- project implemented as planned -few delays in the implementation -excellent cooperation between partners	- substantial delays in the different activities	- project implemented as planned -excellent cooperation between partners
**Capacity to learn, improve and innovate**					
Project collects reliable and valid monitoring data	- credible real time monitoring data collected	- credible real time monitoring data collected	- no disaggregated data collected on attendance rate to the sessions	- credible monitoring data collected	- no credible data collected on home visits
Monitoring is used to learn and improve	-implementation of appropriate changes based on data collected	-implementation of appropriate changes based on data collected	-implementation of appropriate changes based on data collected	-implementation of appropriate changes based on data collected	-implementation of appropriate changes based on data collected
**Sustainability**					
Provides sustained benefit to community	- set up of community of practices in the communities	- set up of community of practices in the communities - low engagement of the community for centers	- limited concrete actions taken by the community to sustain the project	-no prospects of maintaining practices over time - complains of VSLA promotors concerning the lack of financial support	- limited concrete actions taken by the community to sustain the project
Prospects of scale-up beyond GMM2	- first draft of Memorandum of Understanding b/ IRC and the Ministry of the Family, Women and Children (MFFE) for FMD	- first draft of Memorandum of Understanding b/ IRC and the Ministry of the Family, Women and Children (MFFE) for FMD	- scale up strategy unclear at this stage	- scale up strategy unclear at this stage - no sign of government buy-in	- evidence of government involvement (PNN)
**Overall recommendation for Scale up**	**Full**	**Conditional**	**Conditional**	**Conditional**	**Conditional**

Green cells indicate compliance with the evaluation criteria. Orange cells indicate partial compliance with the evaluation criteria. Results based on process evaluation and primary data collection among beneficiaries by IPA independent evaluators.

Table 2 reports the results of the pilot evaluations. Each cell describes the data and information used to evaluate the specific criterium for a given pilot, with cells highlighted in green indicating full compliance with the agreed upon criterium. As the criteria capture a variety of aspects about the pilots, the data sources for each criteria differ, some being more qualitative in nature while others are quantitative (see Annex B1 for details). The criteria to evaluate sustainability, for instance, rely heavily on qualitative key informant interviews. The criterium related to achieving the targeted outcomes in terms of ECD knowledge and parenting behavior, on the other hand, is based on the independent quantitative data collections at baseline and endline. Scoring on this criterium is based on a before-after comparison of beneficiaries’ answers to questions capturing knowledge and parenting practices covering the different topics of the respective curriculums (see Supplementary Table SA1). The evaluation during the pilot stage was hence based on documenting change, with more rigorous evaluation methods to estimate causal impacts reserved for the scale-up. See materials and methods section for detailed results on targeted indicators for each pilot.

The table shows large heterogeneity in results between pilots and illustrates that while some criteria - notably positive feedback by beneficiaries as well as good use of monitoring data for improvements in implementation–were fulfilled by all pilots, other criteria were more difficult to achieve. Particularly notable were doubts on the sustainability of benefits in target communities, with 4 out of 5 pilots containing components for which it was deemed unlikely they could be maintained after the end of the pilot period. Moreover, for 3 out of 5 pilots, partners had an incomplete vision on cost management under a scaling scenario. We will return to these points in the conclusion.

The first pilot in Table 1 with all 11 criteria compliant received an unconditional recommendation for scale-up. As this pilot included trainings by highly qualified ministry staff (social workers of the Ministry of women, family, and children, MFFE) to relatively large groups of beneficiaries (25) for a two-hour weekly session over 11 weeks we will refer to this pilot as the “intensive group training” pilot. The remaining pilots received a conditional recommendation, with conditions being corrective actions addressing the partially compliant criteria. As a result, TRECC and its partners decided to scale the core component of the “intensive group training” pilot. While pilot 1 also included the construction of preschools, the donors and the government conjointly decided that this component would not be systematically scaled in parallel, as they could not secure the financial and human resources necessary for their sustainability.

While none of the other pilots was compliant on the 11 criteria, given the prospects for scale-up through the existing structures of MSHP (Ministry of health and public hygiene), the parental training approach tested in the 5th pilot (Table 1) was further adapted. As this pilot relied on cascade training with knowledge transfer from ministry staff, to paid community health workers, to community volunteers and then to parents, we refer to this pilot as the “cascade training pilot.” Notably, this pilot, implemented by two partners with strong institutional expertise in nutrition and health, had shown positive change for nutrition but not for child stimulation. An adjustment phase in four new pilot localities therefore tested a revised curriculum for stimulation based on (26), new learning methods that stimulated interactive discussions, and improved training for community health workers. The adjustment phase also helped identify remaining constraints around the motivation of volunteers and beneficiaries with formative research pointing to barriers to participation and change in ECD practices (27–29). Certain training topics (such as using positive disciplining methods rather than physical punishment; or the age at which to introduce solid foods into children’s diets) were found to be less well accepted because of existing beliefs and cultural norms, resulting in community pressure on parents deviating from social norms. Qualitative findings further revealed misconception around certain practices and pointed to lack of buy-in by fathers as reasons for low participation. Approximately 40% of session for mothers and almost 50% of session for fathers and grandmothers had participation rates lower than 80%, implying that a substantial share were missing out on key messages (Supplementary Table SA2). Grandmothers were found to play a key role in transmitting ECD practices across generations. These resonate with other evidence from Sub Saharan Africa (30–36).

## Rigorous testing of interventions at scale

The formative research on the pilots led to the identification of several questions in need of rigorous evidence prior to national scale-up. Two RCTs were set up to test the cost-effectiveness of the two core models selected for scaling (see Materials and Methods section B for details on the RCT designs and study protocols) through a process of co-design between the various partners and an independent research team. While the trials are not at population scale, they were designed to be scalable, as in (6).

The Minister of Women, Family and Children and the NGO IRC (International Rescue Committee) teamed up to roll-out the intensive group trainings in 158 villages spread across 5 departments (Agneby Tiassa, Goh, Lôh-Djiboua, Haut Sassandra, and Nawa). Following the approach from pilot 1, the projects targeted the main caregiver through formal in-group training sessions, with 25 beneficiary households with children 0–5 years old per locality, selected among members of the Village Savings and Livelihood Associations (VSLAs). The trainings covered the FMD (“Families Make a Difference”) curriculum over a period of 11 weeks (one per week). Trainings were given by local social workers employed and identified by MFFE. Each social worker was assigned two villages, to allow them to combine the training sessions with their other assigned tasks and responsibilities. Social workers are all trained in community-level training activities and are employed in a variety of such activities as part of their regular responsibilities. The FMD curriculum has a strong focus on (verbal) communication, positive discipline, and intra-household relationships, and encourages the use of home-made toys rather than material inputs.

The scaled-up version of the “cascade training model” from the 5th pilot, implemented by the National Program for Nutrition (PNN) and the NGO HKI (Hellen Keller International) targeted all households with children 0–5 years old in 173 different villages, covering the universe of villages in the sanitary district of Lakota (in the department of Lôh-Djiboua) through in-group training sessions and home visits. Monthly group training sessions were offered for a period of 10 to 15 months, to groups of 15 caregivers at a time, adapted from the “Care for Child Development” program. This was complemented with home visits (1 h, once a month for each household) and village-level sensibilization activities. Trainings and home visits were the responsibility of community-volunteers, trained and supervised by community health agents, who report to the local health district personnel of MSHP (nurses or pharmacist), and receive technical backstopping from HKI. Ministry staff was also responsible for initial community mobilization. Both community volunteers and community health agents were identified for this project specifically and have no responsibilities outside of the project. The local health district personnel, however, combine the oversight tasks with their regular responsibilities. The training integrates topics on early childhood learning and stimulation, positive discipline and socio-emotional support with nutrition and hygiene messages.

**Figure 2:**
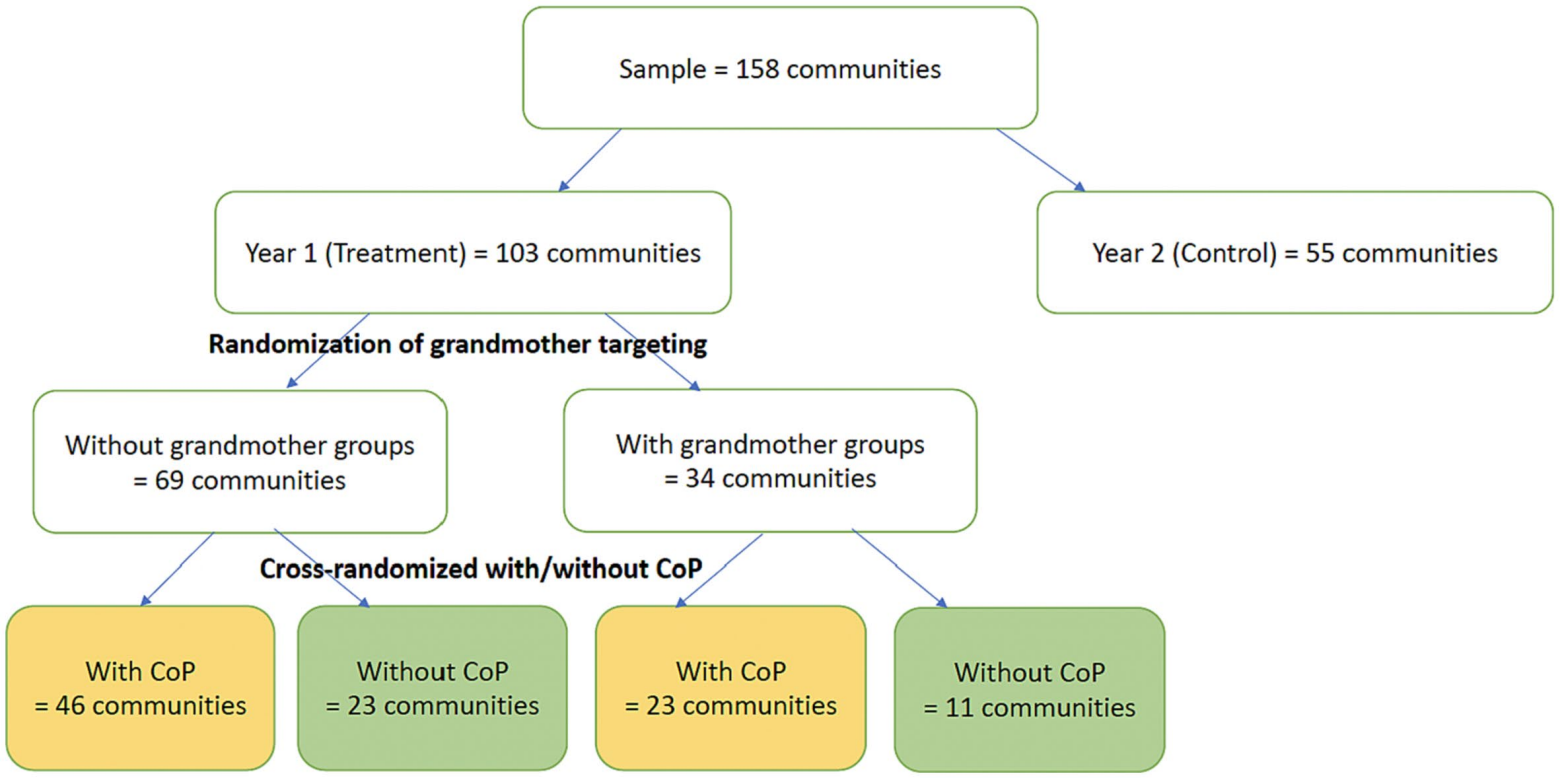
Design of the experiment on the intensive group training project.

**Figure 3:**
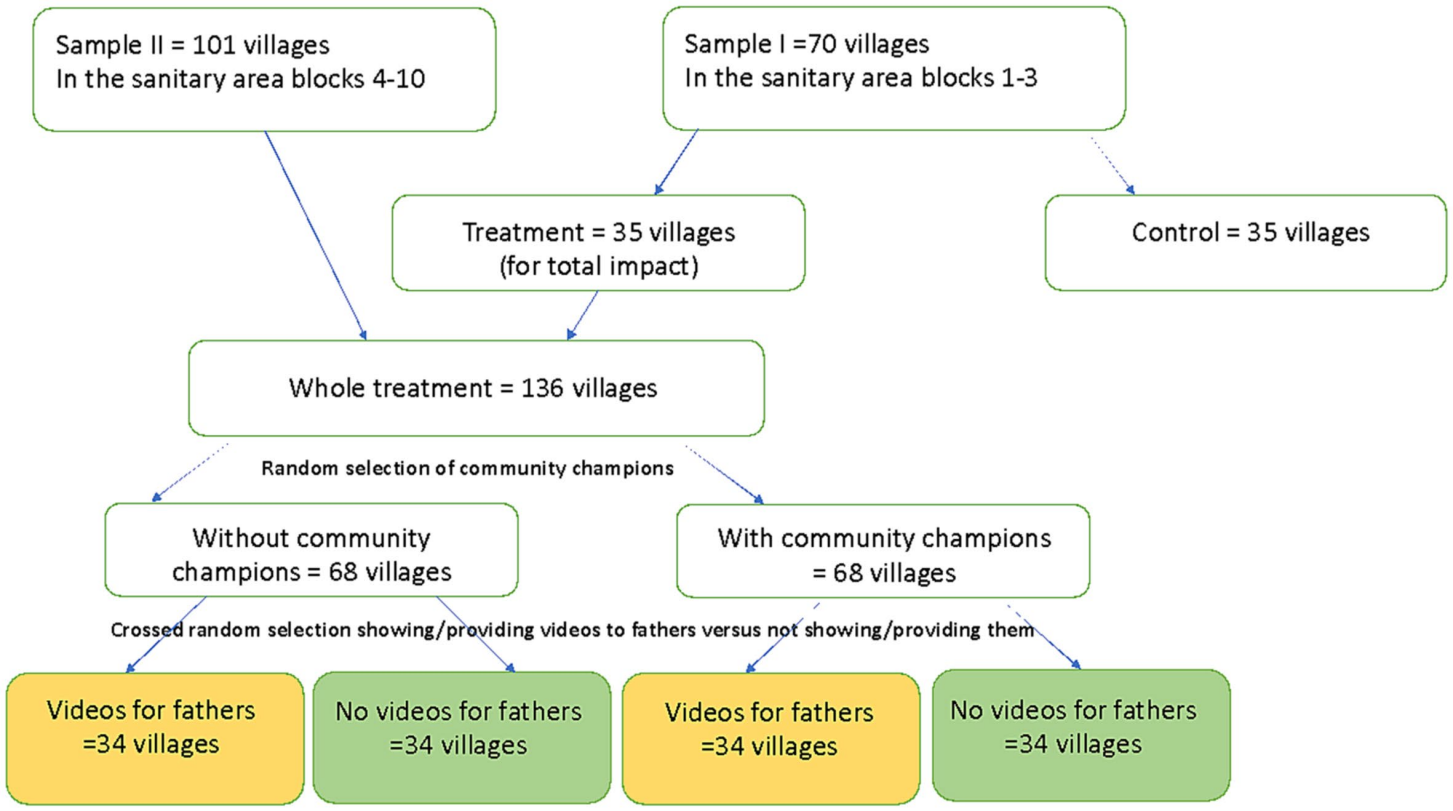
Design of the experiment on the cascade training project.

Apart from testing the cost-effectiveness of the two models, the RCTs also aim to test whether the effectiveness depends on interventions’ ability to sustainable shift intrahousehold and community social norms around ECD. Randomized variations were introduced to estimate the differential impacts of additional training sessions for grandmothers, raising fathers’ awareness through targeted videos, setting up Communities of Practices (CoP) around ECD allowing a larger group of parents to be exposed to the training messages, and seeking the active involvement of selected community champions (Figures 2, 3). The community champions are members of the village leadership or other influential figures in the community, who were invited to the training by the ministry staff and then asked to help mobilize and motivate parents for the trainings by the volunteers. Training started in August 2021, along with data collection to measure impacts. Cost data using the ingredient approach (37) were collected in parallel.

The two approaches that are being tested strongly rely on existing infrastructure, and in particular human resources and ministry staff, but under two different models of knowledge transmission. Both models relied on local ministry staff with tertiary education relevant to the topics of the parental training, which was deemed important by the implementing partners for the initial transmission of the trainings’ messages. As such ministry staff is locally relatively scarce, their time commitment provides a potential important constraint on scalability. It is therefore useful to compare the models from this perspective (Supplementary Figure SA1). The intensive group training model relies on direct training by MFFE ministry staff, each of whom provides training in two villages, with one NGO staff providing light-touch technical assistance covering 40 villages. In contrast, the model followed by PNN and HKI has a much stronger reliance on cascade training. Each ministry staff supervises about 3 community health agents, who in turn train and support 6.5 community volunteers on average. The NGO staff provides technical assistance to ministry staff and community health agents (covering about 20 villages). In the intensive group training model, ministry staff directly train a large share of targeted households (25 per village) while another 15 households per village are then reached through the CoP. With full compliance, this comes down to 7 households trained per day of ministry staff’s time. In contrast, in the cascade training model 1 day of ministry staff indirectly helps reach approximately 20 households, though with all training of parent beneficiaries done by (much) lower educated community volunteers. Apart from possible implications on the quality of the knowledge transmission, the extent to which either model leads to impacts on parental behavior and, ultimately on children’s outcomes will also depend on participation to trainings by targeted beneficiaries, to which we turn next.

## Take-up, acceptability, and retention of key messages during scale-up

As for the pilot, the analysis of the effectiveness of the two models implemented at scale rely on a combination of monitoring data collected by the implementing partners, and independent data collected by IPA through qualitative and quantitative surveys (see Annex B3). Analysis was done by the authors of this article.

The monitoring data shows that participation in the intensive group trainings was high, even without financial compensation to parents, with 73.6% of beneficiaries attending at least 80% of sessions (median rates of participation is 90%). Participation remained stable over the course of the intervention (Supplementary Figure SA2). Attendance records show no differences between experimental variations in take-up, suggesting that the core design was sufficient to assure high participation by parents, with no additional gains in take-up of the main beneficiaries from targeting grandmothers or CoPs (See Supplementary Table SA2). Results confirm the broad reach of the trainings among the 25 pre-identified households, with take-up highest among parents with more children, echoing (38).

While the continued high take-up over 11 weeks is suggestive, we further analyze acceptability through information from a survey on beneficiary parents conducted just after the trainings. Supplementary Figures SA3a,b show large retention of the key messages, with virtually all mothers and grandmothers remembering at least one message, and messages about communication with children, feeding and disciplining mentioned by many. While about 30% of mothers (60% of grandmothers) point to at least one message that is hard to implement, a very large share (82% of mothers and 83% of grandmothers) report agreeing with all messages and almost none of the caregivers reported lack of agreement with messages as a reason for having missed sessions. An IRC satisfaction survey confirms the positive feedback, with both main beneficiaries and grandmothers reporting they found sessions useful, enjoyable, clear, and understandable. These results provide support for a key assumption underlying the Theory-of-Change, as the general interest and receptibility for key messages is a precondition for further impact.

A similar satisfaction survey for the cascade trainings in the PNN-HKI scale-up project was also positive, with more than 90% of respondents indicating that content was interesting, easy to understand and helpful in daily life. Even so, available data from the first 5 months of trainings show that participation rates to training sessions declined compared to the pilot, with 65% of sessions having an attendance rate lower than 80%, and a median attendance rate of 60%. Interestingly, however, administrative data on attendance show higher participation in organized sessions in villages where fathers were randomly exposed to the videos, and also in villages randomly selected to have community champions. Notably, father’s video exposure leads to higher participation of mothers (Supplementary Table SA4). Mobilization by community champions increased the number of mothers per session by 2.36 (CI: 0.49 to 4.22; *p* = 0.014) while the videos increase participation on average by 1.78 (CI: −33 to 3.90; *p* = 0.097). The effectiveness of the community champions and video exposure to increase participation is confirmed with attendance data from the home visits. Participation of fathers in home visits goes up with 17 percentage points (p.p) with video exposure (CI: −0.00 to 0.34: *p* = 0.059), while participation of mothers goes up with 10 p.p (CI: 0.03 to 0.18; *p* = 0.005). Community champions increase mothers’ participation with 6 p.p. (CI: −0.00 to 0.12; *p* = 0.060). These results hence provide supporting evidence for the importance of intrahousehold and community dynamics to increase take-up of parental training, suggesting openness of mothers to the interventions in part can depend on perceived acceptability of the messages by their husbands and the wider community.

Openness to the intervention and its messages of course does not mean that they have been internalized, or that they lead to the same behavioral changes in the two interventions or across the variations. There is also an open question on whether all messages were effectively passed on through the cascade models of learning that both interventions rely on. Results from tests conducted on volunteer trainers of the cascade model before and after the training-of-trainers suggest this could be concern, as their knowledge only increased by 50% compared to that of the trainers-of-trainers (Supplementary Table SA5). Hence while the experimental evidence-to-date points to mechanisms to increase reach and acceptability at scale, impacts on final outcomes will need to be studied as the RCTs are completed.

## Discussion

The iterative and multi-partner process of small-scale piloting, monitoring, evaluation, formative research, adaptation, and subsequent testing at scale has led to two models of parenting training that have wide acceptability. Participation by the intended beneficiary parents differed between the two models, possibly reflecting different targeting. The primary target of the intensive group training intervention are 25 pre-identified parents who were organized in VSLA and had expressed interest in parenting training, while the CoP are meant to subsequently reach a wider set of households. In contrast, the cascade training model set out to reach all households with children 0–5 in a village simultaneously. In this later model, parental participation was found to be affected by community and household influencers. Even so, overall participation stayed relatively low, and imperfect knowledge transmission through the cascade also raise questions regarding its sustainability.

For parenting interventions to have impacts at scale, complying with early steps in the theory-of-change, including high-quality implementation of trainings, take-up by parents and acceptability of messages are important preconditions. The iterations and learning cycles of the various pilots and scaled up versions in Côte d’Ivoire provided an opportunity to learn across different implementation models on those early steps. One particularly salient trade-off highlighted by the two models being tested is between recruiting local community-based volunteers and outside trainers. Intense training over a relatively short period by highly qualified trainers, who can rotate to other villages when finishing in a first set, can be one potentially mechanism to limit costs while assuring high quality knowledge transmission. Volunteer-driven models have lower cost per beneficiary and allow to scale to more parents with involvement of relatively few professional staff. They might not be more cost-effective, however, if it is difficult to find volunteers with sufficiently high qualification and motivation to effectively transmit knowledge to parents. Volunteers’ knowledge was found to be relatively low and knowledge transmission was imperfect, risking key messages of the training to become distorted. The evaluations also point to difficulties of maintaining volunteer commitment but provide promising indications of the potential of increasing reach by obtaining buy-in of community and household influencers. The cost-effectiveness analysis of the scaled-up programs will provide quantitative evidence on these different trade-offs to inform national scaling efforts.

As such, the stepwise pilot-to-scale approach employed by TRECC with a strong focus on monitoring, evaluation and learning at every step of the process, has allowed to provide nuanced answers to operational and conceptual questions regarding the scaling of parental training programs, a potential key element of Côte d’Ivoire’s commitment to improving its human capital outcomes. The MEL approach notably has allowed to zoom in on some of the more promising models, and focus energy for adaptation and improvement on those models. Having clearly identified criteria from the start of the piloting process that addressed different criteria to account for when making a scaling decision, and a purposeful and mixed-methods data collection investment specifically designed to evaluate each of those criteria, was undoubtedly a key ingredient to achieving the MEL objectives. The strategy of using both administrative data sources from partners and independent data that could speak to each other, was also crucial for the buy-in of the lessons learned by different stakeholders and partners. Finally, a strength of the TRECC pilot-to-scale approach was to start with multiple models to pilot, which increased the probability to be able to move forward with at least one of them, and as such built in a long-term assurance that there would be a way ahead. Finally, while the multi-partner engagement is costly in coordination, it is crucial for ownership of the results.

Providing comprehensive and actionable information based on common agreed criteria to a certain extent can correct priors of partners, including those with a clear stake in the process. Yet it can be a slow and imperfect process. A clear illustration of this last point was that the interests of several partners led to the scaling of the cascade-training approach even if the MEL information pointed to the remaining weaknesses in the approach. Several stakeholders saw the cascade-training as a necessity, given lack of sufficient staff capacity and technical expertise to eventually scale nationally, and wanted to test the adaptations made as part of the adjustment phase in an “at-scale” implementation. As such, after 5 years of such piloting and adaptation, questions remain on the effectiveness of knowledge transmission in cascade models and on the sustainability of approaches based on community volunteers, pointing to the need for continued learning and adjustments. Addressing those limitations after a scaling decision is made can be difficult, as operational bandwidth constraints and lack of budget flexibility can become more binding when programs are taken to scale. Pilot-to-scale approaches based on rigorous MEL systems to improve ECD outcomes may want to consider flexibility in timelines and accept the possible need for multiple rounds of adaptations, including decisions to delay scale, to maximize returns to the MEL investment.

Finally, because the ultimate payoffs of investment in early childhood only materialize 20 to 30 years after the investment, having reliable, scalable, and effective MEL systems built into ECD programs is crucial for accountability, and therefore ultimately for their sustainability. Retaining buy-in of implementing partners, as well as those parties possibly interested in providing financing for ECD interventions (as is the case with the private sector cacao partners in Côte d’Ivoire), can be facilitated with credible data systems that allow to track fidelity of implementation. Finally, at the appropriate stage and after implementation constraints have been addressed, providing rigorous causal estimates on early childhood cognitive and socio-emotional outcomes becomes crucial to assure long-term effectiveness, as such early childhood outcomes are the best predictors of pay-offs later in life. Future research will therefore return to this question.

## Data availability statement

The raw data supporting the conclusions of this article will be made available by the authors, without undue reservation.

## Ethics statement

The studies involving human were approved by Paris School of Economics Institutional Review Board and by the Comité National d’Éthique des Sciences de la Vie et de la Santé (CNESVS) of Côte d’Ivoire. The studies were conducted in accordance with the local legislation and institutional requirements. Informed consent for participation in this study was provided by the participants and by the legal guardians/next of kin for all participants that were minors.

## Author contributions

TF, SG, KM, CP, CT, and JV conceptualized and designed parts of the study. RA, TF, CP, SR, CT, and JV designed part of the data collection instruments and collected data. RA, CP, SR, CT, and JV led part of the data analysis. TF, SG, CP, and CT drafted parts of the initial manuscript, and reviewed and revised the manuscript. RA, SR, and JV critically reviewed the manuscript for important intellectual content. KM coordinated and supervised part of the design of data collection instrument and data collection, as well as all data analysis, drafted the initial manuscript, and reviewed and revised the manuscript. All authors contributed to the article and approved the submitted version.

## Funding

The research is funded by TRECC: an initiative funded by the Jacobs Foundation, Bernard van Leer Foundation and UBS Optimus Foundation and the French National Research Agency (ANR) under grant ANR-17-EURE-0001.

## Conflict of interest

The authors declare that the research was conducted in the absence of any commercial or financial relationships that could be construed as a potential conflict of interest.

## Publisher’s note

All claims expressed in this article are solely those of the authors and do not necessarily represent those of their affiliated organizations, or those of the publisher, the editors and the reviewers. Any product that may be evaluated in this article, or claim that may be made by its manufacturer, is not guaranteed or endorsed by the publisher.

## Abbreviations

CoP: Communities of Practices
ECD: Early Childhood Development
HKI: Hellen Keller International
IPA: Innovation for Poverty Action
IRC: International Rescue Committee
M&E: Monitoring and Evaluation
MFFE: Ministère de la Femme, de la Famille et de l’Enfant
MSHP: Ministère de Sante et Hygiène Publique
NGO: Non governmental organization
PNN: Program National de Nutrition
VSLA: Village Savings and Loan Associations
