# Supplementary material for: Piloting, testing and scaling parental training: a multi-partnership approach in Côte d’Ivoire
Source: Front Public Health. 2023 Aug 15;11:1106565. doi: 10.3389/fpubh.2023.1106565 (PMC10466389; doi:10.3389/fpubh.2023.1106565)
Supplement: Supplementary file 1 [file Data_Sheet_1.pdf]

Materials and Methods for
Piloting, Testing and Scaling Parental Training: A multi-partnership approach in Cote d'Ivoire

A: Further information on TRECC's approach and its partners

B: Methods
1) Pilot evaluations against 11 criteria
2) Experimental design of the 2 RCTs
3) Data
4) Empirical specification for take-up analysis
5) IRB approval
6) Data availability and replication

Supplementary Tables

Supplementary Figures

A: Further information on TRECC's approach and its partners

The TRECC program aims to improve the living conditions of children and youth in Côte d'Ivoire through improved access to early childhood development services and quality education. To achieve this goal, TRECC's theory of change consisted in the provision of solid evidence as well as rigorous process evaluations that are used by the government and other key stakeholders to strengthen public policy and enhance ECD programs design. Two main components stem from TRECC's approach:

1. **Building an ecosystem of stakeholders and catalyze collective action and resources for augmented sustainable impact**

In essence, issues related to child development are complex, multidimensional and multisectoral, and require responses that tap into collective efforts. This means shifting away from organization individual projects paradigm to pooling resources and expertise for systemic change.

With this in view, TRECC has mobilized a range of diverse stakeholders beyond organizations and individuals traditionally involved in early childhood development. TRECC has brought together the Government of Côte d'Ivoire, philanthropic foundations, cocoa and chocolate companies, the civil society, national and international NGOs, and academic partners to form an innovative ecosystem of stakeholders with the common goal of providing every child with a good start in life and the opportunity to develop their full potential.

With this approach, TRECC has fostered innovative Public-Private partnerships with the government and the cocoa and chocolate industry as key stakeholders to explore new ways of addressing physical, cognitive and socioemotional delays in early childhood to inform public policy but also achieve sustainability strategies of the cocoa industry.

In this framework, TRECC launched its Grant-Matching Mechanism (GMM) to catalyze an initial investment of 10 million CHF by the cocoa and chocolate industry in child learning and development.

2. **Building evidence for sound policy and decision making**

Solid evidence about what works to improve outcomes for children under which conditions, combined with high-quality metrics on results is critical for effective allocation of resources and providing contextualized actionable solutions. Evidence generation and data-driven decision-making are at the core of TRECC's interventions. Since its inception, TRECC has adopted a pilot to scale approach with the development and implementation of a research and learning agenda throughout the scaling process. Experience shows that social sector innovations almost never scale spontaneously. Scaling successful interventions is rather the result of a deliberate process, requiring a robust scale-up plan, strong commitment from key stakeholders and iterative adaptation during the implementation phase. Along this process, TRECC has launched 5 pilot-to-scale ECD projects, implemented by selected local and international NGOs, built upon existing evidence-based models proven to be effective at improving ECD outcomes at affordable costs, relevant to the local context with a history of replication. The purpose of the pilot phase was to adapt and test these approaches in the context of rural Côte d'Ivoire and assess their potential for integration in national policies and programs, as well as in corporate sustainability strategies of cocoa and chocolate industry members.

The 11 criteria on which the pilots were evaluated aimed at testing their scalability based on whether:

- Implementation can be based on volunteers or executed by local organizations
- The intervention approaches can be institutionalized, mainstreamed into existing government systems (as scaling vehicle) to ensure engagement and sustainability of actions (vertical scaling)
- The intervention areas can be increased to reach more communities, parents, and children (horizontal scaling)
- Capacity building and strengthening of key stakeholders is feasible, particularly government officers. (Organizational scaling)

TRECC mandated Innovation for Poverty Action (IPA) to provide technical support to the companies and implementing organizations throughout the pilot phase to ensure sound monitoring systems allowing for regular data collection and learning from these projects. IPA also conducted its own independent and complementary data collection to evaluate the pilot projects. For each pilot, IPA analyzed these two sources of information—the data collected regularly by the implementing organizations through their own monitoring system and IPA's independently collected data—to assess the pilot on a set of criteria including the relevance of the project, the achievement of planned results, the management of costs and operations, the capacity to learn, improve and innovate and the prospects for sustainability and scale. Only those pilots sufficiently addressing each set of criteria were eligible for an extension phase.

Based on the evaluations of these pilots, two parenting models received positive recommendation for the extension phase prior to full scale-up. The extension phase is unfolding along with a robust embedded impact evaluation to better understand the cost-effective approach that can contribute to achieve the scale up ambition. Results of the cost-effectiveness study aim to inform a wider investment of 40 million US$ under new Early Learning and Nutrition Facility ELAN, that is to be placed under the leadership of the CONNAPE to finance ECD interventions of the different line ministries (MFFE and MSHP).

List of partners

National Government

MFFE :
Ministère de la Femme, de la Famille et de l'Enfant - Ministry of Women, Family and Children

SCESCS:
Service de Coordination des Structures Educatives de Base du MFFE

MSHP
Ministere de Sante et Hygiene Publique, Ministry of Health and Public Hygiene of Côte d'Ivoire

PNN:
Programme national de Nutrition PNN – National Nutrition Program of MSHP

PMNDPE
Projet multisectoriel de nutrition et de développement de la petite enfance
Multisectoral Nutrition and Child Development Project

CONNAPE
Conseil National de la Nutrition de l'Alimentation et du Developpement de la Petite Enfance
National Council for Nutrition, Food and Early Childhood Development

International NGOs
HELEN KELLER INTERNATIONAL (HKI)
A global health organization dedicated to eliminating preventable vision loss, malnutrition, and diseases of poverty. HKI's work cuts across nineteen countries and focuses on a set of problems at the intersection of poverty and human potential. HKI's work with TRECC focuses on nutrition and early childhood development.

INTERNATIONAL RESCUE COMMITTEE (IRC)
A neutral international humanitarian organization. IRC's mission is to help people affected by crises to survive, recover and rebuild their life. IRC opened its offices in Côte d'Ivoire in 2003 to help the Ivorian population in the early hours of the political crisis. In Côte d'Ivoire, IRC works in close collaboration with civil society, local authorities and the beneficiary population and carries out many social actions within the framework of its programs of Protection and Development of children and youth.

INTERNATIONAL COCOA INITIATIVE (ICI)
A non-profit foundation that works to protect the rights of children and adults in cocoa-growing areas in West Africa. ICI's vision is of thriving cocoa-growing communities within a dignified, sustainable and responsibly managed cocoa supply chain, where child rights and human rights are protected and respected, and where child labour and forced labour have been eliminated.

RIGHTS EDUCATION AND DEVELOPMENT CENTRE (READ)
A non-profit organization. READ's approach combines education, enterprise and community development to empower people living in rural communities to thrive and prosper, using READ Centers as its platform. READ Centers are designed to serve whole communities. READ did not have an office/staff in Côte d'Ivoire and provided remote support to the TRECC pilot project.

THE INTERNATIONAL RESEARCH & EXCHANGES BOARD (IREX)
An international non-profit organization providing leadership and innovative programs to improve the quality of education, strengthen independent media, and foster pluralistic civil society development. IREX has extensive experience taking proven community development models and adapting them to new contexts, particularly in complex settings. IREX did not have an office/staff in Côte d'Ivoire and provided remote support to the TRECC pilot project.

CARE
A humanitarian organization which began working in Côte d'Ivoire in 2000. CARE started implementing VSLA model in Cote d'Ivoire in 2007 and since then has improved and adapted the model to be fully gender transformative, providing a strong enabling environment

for women to thrive socially and economically in stable groups that can tackle any social issue important for the advancement of the women empowerment agenda.

STRENGTHENING FAMILIES PROTECTING CHILDREN (ICS)
An East African NGO that works in rural areas of Africa together with families, communities, local and national government and other stakeholders to create safe and nurturing environments for children to reach their full potential. At the core of its work is Skilful Parenting and Ending Violence against Children. These are embedded in well-targeted economic empowerment and agribusiness programmes that aim to improve household income and food security. ICS had no staff in Côte d'Ivoire at the time of the pilot.

Financial partners

TRECC
Transformer l'Éducation dans les Communautés de Cacao - Transforming Education in Cocoa Communities program - is an initiative funded by the Jacobs Foundation, the Bernard van Leer Foundation and UBS Optimus Foundation.

JACOBS FOUNDATION
invests in the future of young people so that they become socially responsible and productive members of society. In order to achieve this goal, children and youth must be given better opportunities for positive development and equitable access to education.

BARRY CALLEBAUT (BC-SACO)
a Belgian-Swiss chocolate manufacturer.

TOUTON
a French trading company. The group is a large trader in the world of cocoa, and more generally, one of the main trading groups in tropical agricultural commodities.

MONDELEZ
an American multinational food company, particularly active in the cookie and chocolate sectors, with operations in many countries around the world.

MARS
an American food company and agribusiness group with operations in about 100 countries.

BLOMMER
an integrated chocolate manufacturer and chocolate-ingredient supplier based in Chicago, Illinois.

HERSHEY Company
commonly known as Hershey's, is an American multinational company and chocolate manufacturer.

Research Partners
INNOVATIONS FOR POVERTY ACTION (IPA)

a research and policy nonprofit that discovers and promotes effective solutions to global poverty problems, working closely with researchers and decision-makers to design and test development programs.

PARIS SCHOOL OF ECONOMICS (PSE)
a French research institute in the field of economics.

BUSARA Center
a Behavioral Science organization headquartered in Kenya that believes in a world where evidence-based and context-specific solutions are routinely implemented to address the world's most pressing problems.

CENTER FOR COMMUNICATION PROGRAMS (CCP)
a center on social and behavior change at Johns Hopkins University Bloomberg School of Public Health

Other

DMI
a social enterprise set up to bring scientific rigour to mass media campaigning by modelling how to save the most lives, designing engaging campaigns and testing their impact, headquartered in the UK.

B: Methods

B1. Pilot evaluations against 11 criteria

IPA collected baseline and endline data for each pilot. The baseline survey characterizes the situation before the intervention and was used to analyze the pilots' targeting, and in particular to evaluate whether the program's selection criteria led to the inclusion of households with highest needs as beneficiaries (criterium 1). The endline surveys interviewed beneficiaries to understand whether and how the intervention affected them and collected their feedback about the program (criterium 5). To assess if targeted direct outcomes (criterium 4) changed in the period of the pilot, questions on knowledge and practices were asked to the main caregivers (and hence beneficiary) in the household at baseline and endline. This data is used for a before-evaluation evaluation. The questions on knowledge were asked to the entire sample of beneficiaries, while age-specific questions for practices were asked to beneficiaries who cared for children in the relevant age range. Questions on children's outcomes in various developmental domains were not included, given the short period between baseline and endline and small sample sizes.

For most pilots, this data is complemented with one or two surveys on the same respondents during implementation (follow-up or spot check visits). Combined with the partners' administrative data – these provide information on the activities, outcomes, and the learning process during implementation (criteria 3,6,7,9,10)

IPA agents collected quantitative and qualitative data through individual surveys, focus groups, key informant interviews, and direct observations.

The costs, with the largest cost category being personnel costs, are estimates of the monetary costs per child on pilot-scale. They are similar in magnitude to the cost estimates in published work on parenting interventions [31]. They are however not representative of expected costs at scale, and therefore mostly presented for completeness. Importantly, the pilot costs as such were not considered in the evaluation criteria. Instead, a key consideration for the scale-up decision was that both models leverage some of the key components of their intervention to reduce cost at scale. For instance, the CoP mechanism helps in reducing cost per beneficiary and help reach a larger number of community member (economies of scale). For the cascade training intervention, the approach based on volunteers can be cost-effective if their motivation and commitment is reinforced and sustained.

B2: Experimental design of the two RCTs

Two randomized control trials were conducted to measure the impact of two interventions and of the different experimental modalities. The two clustered RCTs take advantage of the planned roll-out and scale-up of the two most promising approaches for parental training identified from earlier pilot work. The government and its partners are testing two main intervention designs, and two experimental variations in design in each of them. Figure 1 and 2 summarize the experimental design.

The first is a partnership between SCESCS (under the Ministry in charge of women, family, and children affairs (MFFE)), and IRC that offers group training sessions on the FMD ("Families Make a Difference") curriculum to 25 caregivers in 103 randomly selected

villages (out of 158) for a period of 11 weeks (one per week), with the remaining 55 being phased in after a year. Trainings are given by local social workers employed and identified by MFFE. Each social worker was assigned two villages, to allow them to combine the training sessions with other assigned tasks and responsibilities (which include preschool or special-needs teaching, various types of community sensibilization, and related local social service prevision). Social workers are all trained in community-level training activities and are employed in a variety of such activities as part of their regular responsibilities. The FMD curriculum has a strong focus on (verbal) communication, positive discipline, and intra-household relationships, and encourages the use of home-made toys rather than material inputs.

In 67 of the 103 villages, the 5 most participative caregivers were invited to create Communities of Practice to disseminate their learnings to other parents not selected for the formal training. By only offering formal training to a subset of parents in each village and by fostering informal sharing of information, the design allows to identify indirect impacts of the program through community-sharing and how these information-sharing ties can be used to increase cost-effectiveness. Through an orthogonal randomization, in half of the 103 villages additional slightly adapted trainings are offered to elderly women from the beneficiaries' families, believed to be enforcers of social norms around parenting to test whether this can effectively increase the programs' impacts on household behavior towards young children. The grandmother's curriculum is based on the main caregivers' curriculum: it covers all the same topics but in a simplified way.

The second intervention is implemented through partnership with PNN (under the Ministry in charge of Public Health and Hygiene (MSHP)) and HKI, offering monthly group training sessions to groups of 15 caregivers at a time, adapted from the "Care for Child Development" program. Refresher trainings follow for another 10 months. This is complemented with home visits (1 hour, once a month for each household), conducted by the same community agent who leads the in-group training, and village-level sensibilization activities. In the first 10 months of the program, 133 out 169 villages are being gradually phased in to receive the treatment. Specifically, the project is rolling out activities in blocks of 3-4 sanitary areas (each comprising about 17 villages). For the first 3 blocks, we randomly selected half of the villages in each of the selected sanitary areas as control villages (36 villages). They will be phased in after 10 months, while the first treatment villages will start refresher trainings at that same point.

In half of all treatment villages, influential community members are invited to the trainings, encouraged to discuss their ideas and concerns and assigned the role of Community Champions, to test whether they help shift social norms. The method to identify community champions was first tested through small-scale prototype testing by the implementing partners, with the support of research partners Busara, CCP and IPA. Through an orthogonal randomization, in half of treatment villages fathers are encouraged to participate by showing them videos and introducing them to the training, to test the importance of father's updating regarding parenting practices, and its impact on household norms and investments.

Trainings are given by community-volunteers, trained and supervised by community health agents, who report to the local health district personnel of MSHP (nurses or pharmacist), and receive technical backstopping from HKI. Ministry staff is also responsible for initial community mobilization. Both community volunteers and community health agents were identified for this project specifically and have no responsibilities outside of the project while

the local health district personnel combine the oversight tasks with their regular responsibilities. The training integrates topics on early childhood learning and stimulation, positive discipline and socio-emotional support with nutrition and hygiene messages.

Assignment of treatment and treatment variations was stratified by geographic units.

B3. Data sources

Evaluation surveys for the 5 pilots

For the **IRC-ICI pilot,** IPA collected data on all beneficiaries and non-beneficiaries living in the pilot communities to assess beneficiary targeting at baseline. The sample included the initial beneficiary population of 240 households, as well as 69 non-beneficiary households. The 240 beneficiaries were all sampled for baseline, follow up and endline, independently of whether or not they continued in the program or dropped out), with endline attrition at 10% (N=216). Baseline data includes measures of 4 domains to construct an early childhood development index (ECDI) defined as follows:
1. Literacy-numeracy: Children are identified as being developmentally on track if they can do at least two of the following: identify/name at least 10 letters of the alphabet; read at least 4 simple, popular words; and/or know the name and recognize the symbols of all numbers from 1 to 10.
2. Physical: If the child can pick up a small object with two fingers, like a stick or rock from the ground, and/or the mother/primary caregiver does not indicate that the child is sometimes too sick to play, then the child is regarded as being developmentally on track in the physical domain.
3. Social-emotional: The child is considered developmentally on track if two of the following are true: The child gets along well with other children; the child does not kick, bite or hit other children; and the child does not get distracted easily.
4. Learning: If the child follows simple directions on how to do something correctly and/or when given something to do, and is able to do it independently, then the child is considered to be developmentally on track in the learning domain.
Response categories for all questions included in the ECDI are yes, no and don’t know. The ECDI score is then calculated as the percentage of children aged 36 to 59 months who are developmentally on track in at least three of these four domains.

For the **IRC-READ-IREX** pilot, IPA collected data on all beneficiaries and non-beneficiaries to assess beneficiary targeting at baseline (N=140), including 113 of total of 200 beneficiaries. At endline, IPA re-interviewed 107 of the baseline survey beneficiaries, corresponding to 98% of the beneficiary sample. Baseline data includes measures of 4 domains to construct an early childhood development index (ECDI) defined as above.

For the **BC-ICS** pilot, IPA conducted a baseline survey on 180 beneficiaries (sampled from total of 307 beneficiaries). At endline, 169 beneficiaries out of the 180 were re-interviewed (attrition 6%).

For the **CARE-ICS** pilot, IPA collected data on beneficiaries (90) and non-recipients to assess beneficiary targeting at baseline. Due to logistical obstacles and delays in project implementation, the actual start of the program occurred five months after the baseline survey, which resulted in many respondents dropping out of the program and being replaced

by new beneficiaries. As a result, only 42 of the 90 beneficiaries interviewed at baseline were re-interviewed at endline.

For the **HKI** pilot, IPA collected baseline data on 320 of the 4531 beneficiaries. At endline 270 of these beneficiaries were re-interviewed for an attrition rate of 15%. Beneficiaries include mothers, fathers and other caregivers. For comparability with other pilots, we report results for mothers (145 at baseline, of whom 119 were re-interviewed at endline).

Take-up data SCESCS-IRC intervention at scale

We have access to individual-level attendance records of the 11 sessions of trainings of the main caregivers, as reported by the social worker giving the training. Trainings were organized in 102 of the 103 selected localities (the one exception being a locality where due to a conflict between ethnic group trainings could not be organized). Records are complete for the 102 localities. In each locality, 25 beneficiary caregivers were identified prior to the start of the trainings, and individual attendance records exists for each of those 25 caregivers. This were used to calculate aggregate attendance per locality. The attendance data was merged using unique beneficiary identifiers with baseline data to analyze to what extent take-up and attendance differ depending on the beneficiary households' socio-economic characteristics (see below). Baseline data of all the beneficiary households were collected during the enrollment phase prior to the start of the training.

Take-up data PNN-HKI intervention at scale

We have access to attendance data of training sessions and home visits organized between August 2021 and February 2022, as reported by the volunteer trainers, who record for each of the sessions and home visit they organized the number of people that attended, together with the date and topic of the session, and whether the session targeted mothers, fathers or grandmothers. As the target number for group trainings of beneficiaries for each volunteer trainer is 15, we calculate attendance rates by dividing the number of people attending by 15. As certain volunteer trainers report more than 15 people (possibly because they combined sessions) this leads to some outliers with more than 100% attendance rate. We therefore report the median attendance rate.

As the roll-out of the intervention is ongoing, the available monitoring data does not cover the full period of implementation. The analysis draws on data from 63 localities. Each volunteer trainer reported on multiple sessions, and localities have multiple volunteer trainers, leading to a total of 1228 sessions with attendance data.

The analysis necessarily only focusses on those volunteer trainers that started training activities and reported attendance, with attrition among trainers or selection into reporting to be analyzed in future research.

Take-up data HKI pilot

We have access to the full attendance records of the pilot, as reported by the volunteer trainers, who record for each of the sessions they organized the number of people that attended, together with the date and topic of the session, and whether the session targeted mothers, fathers or grandmothers. As the target number of beneficiaries for each volunteer

trainer is 15, we calculate attendance rates by dividing the number of people attending by 15. As certain volunteer trainers report more than 15 people (possibly because they combined sessions) this leads to some outliers with more than 100% attendance rate. For the 6 pilot localities with attendance data, there are monitoring data from 100 volunteer trainers. Each volunteer trainer reported on multiple sessions, leading to a total of 623 sessions with attendance data (with attendance data not reported for 77 sessions).

Take-up data IRC pilot

We have access to individual-level attendance records of the 11 sessions of training, as reported by the social worker giving the training, who record for each session they organized which beneficiaries participated or not. Records are complete for four villages. There is no available data on the 11th session for all beneficiaries of one village. When computing attendance rates, we considered that 10 sessions took place in this last locality, contrary to 11 in the others. 200 parents were selected and enrolled to participate to the sessions, 40 participants per community. 93 percent of the individuals present in the final administrative data match with the initial list of beneficiaries selected before the beginning of the program.

Data on acceptability and retention of messages SCESCS-IRC scale-up

In November 2021, just after the training of the main beneficiaries had been finalized, we collected a midline survey interviewing a total of 2686 main beneficiaries in treatment and control localities. As part of this survey, both the main beneficiaries and the grandmothers in treatment localities were asked about their experiences with the training. This includes questions on the key messages from the trainings that they retained (with answers allowing for multiple options). We also asked beneficiaries whether they disagreed with certain messages and whether there were certain messages that were difficult to implement.

Satisfaction survey for PNN-HKI scale-up

In November 2021 IPA's Right-Fit-Evidence team conducted a satisfaction survey among 296 parent beneficiaries in 25 localities that had started training, asking for their subjective assessment about the trainings with regards to their usefulness, interest, ease of understanding and the level of knowledge of the trainers.

Satisfaction survey for SCESCS-IRC scale-up

In December 2021, IRC with the help IPA's Right-Fit-Evidence team conducted a satisfaction survey among 583 parent beneficiaries and grandmothers in respectively 90 and 30 localities that had started training, asking for their subjective assessment about the trainings with regards to their usefulness, interest, ease of understanding and the level of knowledge of the trainers.

Data on knowledge tests of trainers

PNN-HKI and SCESCS-IRC shared results of knowledge tests conducted before and after training, as measure of knowledge retention, for the scale-up projects. For the PNN-HKI data

is available on the two types of trainers of the cascade model: trainer-of-trainers and volunteer trainers, while for SCESCS-IRC the trainers are social workers. The HKI data covers the first 4 months of the training-of-trainers in 2021. Tests are not comparable for different trainers, but the same test is administered for the pre and the post tests for a same type of trainer. Data is only available for a (non-random) subset of all trainers. For SCESCS-IRC test scores are not available for 20 agents out of 52 (i.e. 38,5%), because of a change in the questionnaire), while both pre and post tests are available for the other 32 agents. For PNN-HKI, 28 out of the 214 volunteer trainers have not been evaluated at all (i.e. 13.1%). Those with both at least one pre- and post-test represents 53% of volunteer trainers; while 16 out of 38 trainer-of-trainers have a pre and a post test.

B.4. Empirical specification for take-up analysis

Given the availability of individual data, analysis for SCESCS-IRC is done at the household level, with the dependent variables defined as the share of sessions the beneficiary attended (out of the total of 11 sessions). We use the randomized assignment of treatment modalities to analyze whether attendance is significantly different in localities with grandmother sessions or with Communities of Practice, by including treatment variables for each of those variations in the same estimation (given orthogonal randomization). All estimations control for the randomization strata fixed effects, and standard errors are clustered at the locality level.

We separately analyze the conditional correlates of take-up by regressing individual's participation rate in the 11 sessions on a set of socio-economic characteristics, including age, gender, marital status and education of the main caregiver, the number of children in the household (or alternatively the age of the youngest child in the household). All estimations control for treatment variations, the randomization strata fixed effects, and standard errors are clustered at the locality level. See Table A2.

Results show participation does not systematically depend on the experimental variations, nor on education or occupation – confirming that a broad group of parents benefitted. That said, participation is lower for single parents, possibly because their time constraints are more binding. Older parents and parents with more children have significantly higher participation (parents of younger children do too, significant at the 10%), possibly confirming that they perceived higher needs for training.

Analysis for PNN-HKI is done at the session level, with the dependent variable defined as the share of target participants attending (out of total of 15 participants). We use the randomized assignment of treatment modalities to analyze whether attendance is significantly different in localities where fathers were shown videos or where community champions were mobilized, by including treatment variables for each of those variations in the same estimation (given orthogonal randomization). All estimations control for the randomization strata fixed effects, and standard errors are clustered at the locality level. See Table A3

B5. IRB approval

The research was approved by PSE IRB (2020 024) and by the Comité National d'Éthique des Sciences de la Vie et de la Santé (CNESVS), ref 199-20/MSHP/CNESVS-kp.

B6. Data availability and replication

The raw data supporting the conclusions of this article, as well as all program files and their documentation, will be made available by the authors for replication, without undue reservation.

Supplementary Tables

Table A1: Before-after comparison of ECD knowledge and practices for five pilots

Pilot	Topics	Indicator	N	% baseline	% endline	% point change	Significance of change
1. IRC-ICI	Child Protection (knowledge)	% of respondents who can identify 3 or more methods of positive discipline	216	90	88	-2	not significant
1. IRC-ICI	ECD (knowledge)	% of respondents who can identify 2 or more benefits of activities that promote child development	216	76	87	11	*
1. IRC-ICI	ECD (knowledge)	% of respondents who can identify 2 or more stimulations that promotive cognitive, speech, emotional and physical development of children	216	58	74	16	*
1. IRC-ICI	ECD (reported practice)	% of children with whom an adult was engaged in four or more activities to promote learning in the past three days	139	32	47	15	***
1. IRC-ICI	Child Protection (reported practice)	% of respondents who left a child alone/with another child under the age of 10 for more than an hour at least once during the past week	147	14	20	6	not significant
1. IRC-ICI	ECD (reported practice)	% of respondents applying only positive methods of discipline	204	7	9	2	*
2. IRC-READ-IREX	Health (knowledge)	% of respondents correctly identifying at least 3 occasions to wash hands	107	70	85	15	*
2. IRC-READ-IREX	Child Nutrition (knowledge)	% respondents of respondents correctly identifying the age to introduce solids	107	27	73	46	*
2. IRC-READ-IREX	Child Protection (reported practice)	% of respondents who agree or strongly agree that a child needs to be physically punished in order to raise child properly	107	23	9	-14	*
2. IRC-READ-IREX	ECD (knowledge)	% of respondents who can identify 3 or more methods of positive discipline	107	13	15	2	not significant
2. IRC-READ-IREX	ECD (knowledge)	% of respondents who can identify 2 or more benefits of activities that promote child development	107	76	81	5	not significant
2. IRC-READ-IREX	ECD (knowledge)	% of respondents who can identify 2 or more stimulations that promotive cognitive, speech, emotional and physical development of children	107	89	92	3	not significant
2. IRC-READ-IREX	ECD (reported practice)	% of children with who an adult was engaged in four or more activities to promote learning in the past three days	75	27	47	20	***
2. IRC-READ-IREX	Child Protection (reported practice)	% of respondents who left a child alone/with another child under the age of 10 for more than an hour at least once during the past week	69	32	18	-14	**

2. IRC-READ-IREX	Child Protection (reported practice)	% of respondents applying only positive methods of discipline	107	4	6	2	**
3. BC-ICS	ECD (knowledge)	% of respondents that can identify at least 2 stimulating activities (cognitive, speech, emotional, physical)	169	79	93	14	***
3. BC-ICS	ECD (knowledge)	% of respondents that can identify at least 4 out of 8 developmental milestones	169	22	29	7	not significant
3. BC-ICS	ECD (reported practice)	% of caregivers having engaged in 4 or more activities that promote learning and school readiness (in the past 3 days)	71	27	35	8	not significant
3. BC-ICS	ECD (reported practice)	% of caregivers having engaged in 7 or more child-rearing activities (in the past 3 days)	71	42	62	20	***
3. BC-ICS	Child Protection (reported practice)	% of caregivers applying only positive methods of discipline (in the last 30 days)	71	18	29	12	**
3. BC-ICS	Child Protection (reported practice)	% of caregivers who left at least one child alone or with another child under 10 for more than an hour (in the past 7 days)	71	14	18	4	not significant
4. CARE ICS	ECD (reported practice)	% of the caregivers who report having engaged in four or more activities to promote learning and school readiness in the past 3 days	42	40	45	5	not significant
4. CARE ICS	Child Protection (reported practice)	% of the caregivers who used only positive child discipline methods with their children in the past 30 days	42	5	26	21	*
5. HKI	Child Nutrition (knowledge)	% of mothers who can name the correct age for introduction of complementary foods	119	58	72	14	*
5. HKI	Health (knowledge)	% mothers who can name three keys occasions for handwashing	119	79	79	0	not significant
5. HKI	ECD (knowledge)	% of mothers who can name identify at least two ways to stimulate the development of a young child	119	84	83	-1	not significant
5. HKI	ECD (reported practice)	% of mothers engaged in four or more activities to promote learning and school readiness in the past 3 days	119	34	32	-2	not significant

Results based on t-test of both-after comparison. *** p<0.01, ** p<0.05, * p<0.1

Table A2: Participation rates in pilots of intensive group trainings (IRC-ICI) and cascade trainings (HKI-PNN)

Participation statistics	Pilot 1 IRC-ICI	Pilot 5 HKI-PNN		
Level of information	individual	sessions		
Group of beneficiaries	All	Mother	Father	Grandmother
Average rate of participation	95.1%	92.0%	83.4%	89.3%
Median rate of participation	100%	84.7%	80%	81.3%
At least 80% of attendance	97%	59.4%	52.7%	52.5%
Financial compensation	YES	NO		

Note: financial compensation consisted in providing 1000FCFA (equivalent to around 1.8 US$ at the time) to beneficiaries per training session as a compensation for their time and the costs of transportation.

Table A3: Impact of experimental variations and socio-economic correlates of attendance in intensive group trainings (SCESCS-IRC) intervention at scale

	(1)	(2)	(3)	(4)
	Attended at least 80% of training sessions			
CoP treatment	0.0304	0.0222	0.0257	0.0261
	(0.0287)	(0.0275)	(0.0276)	(0.0276)
Grandmother training treatment	-0.0184	-0.0152	-0.0144	-0.0141
	(0.0316)	(0.0306)	(0.0308)	(0.0307)
Number of children in household		0.0222***		
		(0.0053)		
Number of children between 0 and 5 in household			0.0311***	
			(0.0091)	
Age youngest child				-0.0111*
				(0.0057)
Age main beneficiary parent		0.0039***	0.0043***	0.0045***
		(0.0010)	(0.0010)	(0.0011)
Main beneficiary parent is single parent		-0.0539*	-0.0642**	-0.0657**
		(0.0314)	(0.0315)	(0.0319)
Main beneficiary without education		0.0130	0.0164	0.0160
		(0.0389)	(0.0389)	(0.0388)
Main beneficiary completed primary education		-0.0238	-0.0193	-0.0200
		(0.0386)	(0.0387)	(0.0386)
Main beneficiary completed upper secondary school		0.0156	0.0171	0.0164
		(0.0724)	(0.0739)	(0.0756)
Main beneficiary completed tertiary education		-0.0994	-0.0901	-0.0949
		(0.227)	(0.221)	(0.215)
Observations	2,472	2,472	2,472	2,472
R-squared	0.069	0.088	0.085	0.083

Note: household level regressions based on attendance records from monitoring data and household characteristics at baseline. All regressions control for strata fixed effects and gender main beneficiary parent. Omitted category for education is lower secondary school. Standard errors, clustered by locality, in parentheses.
*** p<0.01, ** p<0.05, * p<0.1

Table A4: Impact of experimental variations on attendance in cascade trainings (PNN-HKI intervention) at scale:

	(1)	(2)	(3)	(4)
	Mother	Father	Mother	Father
	Number of attendees per session		At least 80% attendance per session	
Community champions treatment	2.36**	2.38**	.16***	0.14**
	(0.93)	(1.13)	(0.06)	(0.07)
Father video treatment	1.78*	.48	0.12*	0.03
	(1.06)	(1.02)	(0.06)	(0.07)
Observations	637	495	638	495
R-squared	.287	.345	0.211	.260

Note: session level regressions based on attendance records from monitoring data up to February 2022. All regressions control for strata fixed effects. Standard errors, clustered by locality, in parentheses. *** p<0.01, ** p<0.05, * p<0.1

Table A5: Pre-post training test results of trainers of the scaled-up interventions

Intervention	Trainers	Average change from pre to post test (in %)	Median change from pre to post test (in %)	SD (in %)	Total number trainers	% of trainers with available data
SCESCS - IRC	Social worker	23.8	21.3	16.5	52	61.5
PNN-HKI	Trainer-of-Trainers	23.1	25.0	12.0	38	42.1
	Volunteer trainers	11.2	10.0	16.9	189	52.9

Note: Results of knowledge tests conducted before and after trainings, as measure of knowledge retention. Tests are not comparable for different trainers. Data is only available for a (non-random) subset of all trainers (see last column).

Supplementary Figures

Figure A1: Number of localities per agent at different levels of the cascade in scaled-up interventions

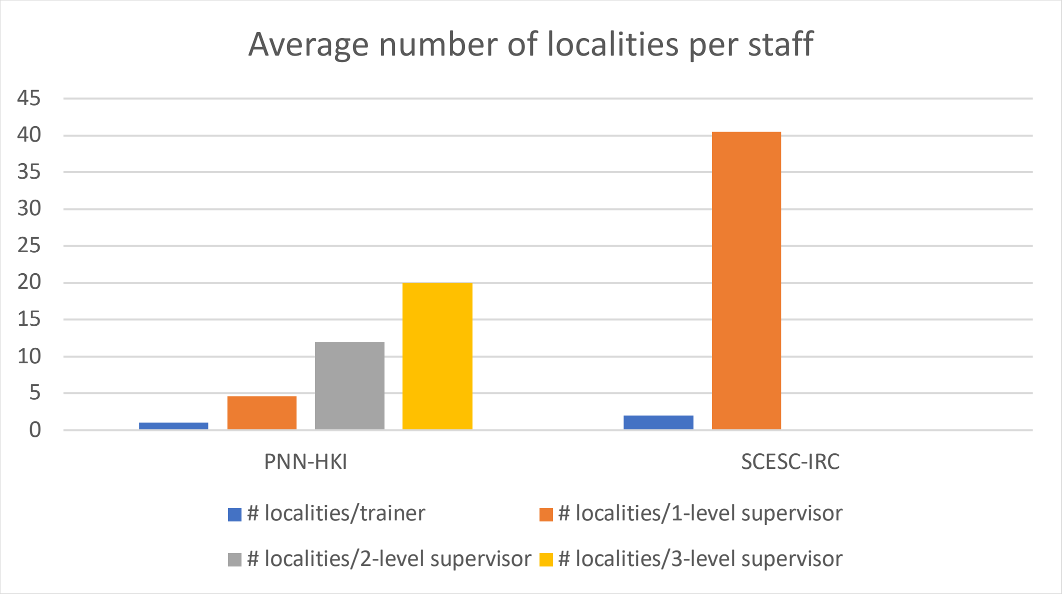

Trainers are volunteers in PNN-HKI (cascade training) and social workers/preschool teachers in SCESC-IRC (intensive group trainings). 1-level supervisor are community health workers in PNN-HKI and NGO technical assistance I SCESC-IRC. 2nd level supervisor are district sanitary agents (nurses or pharmacists) in PNN-HKI. 3rd level supervisor are HKI supervisors providing technical support in PNN-HKI.

Figure A2: Evolution of attendance of main caregiver in intensive group trainings (SCESCS-IRC) scale-up over 11 sessions

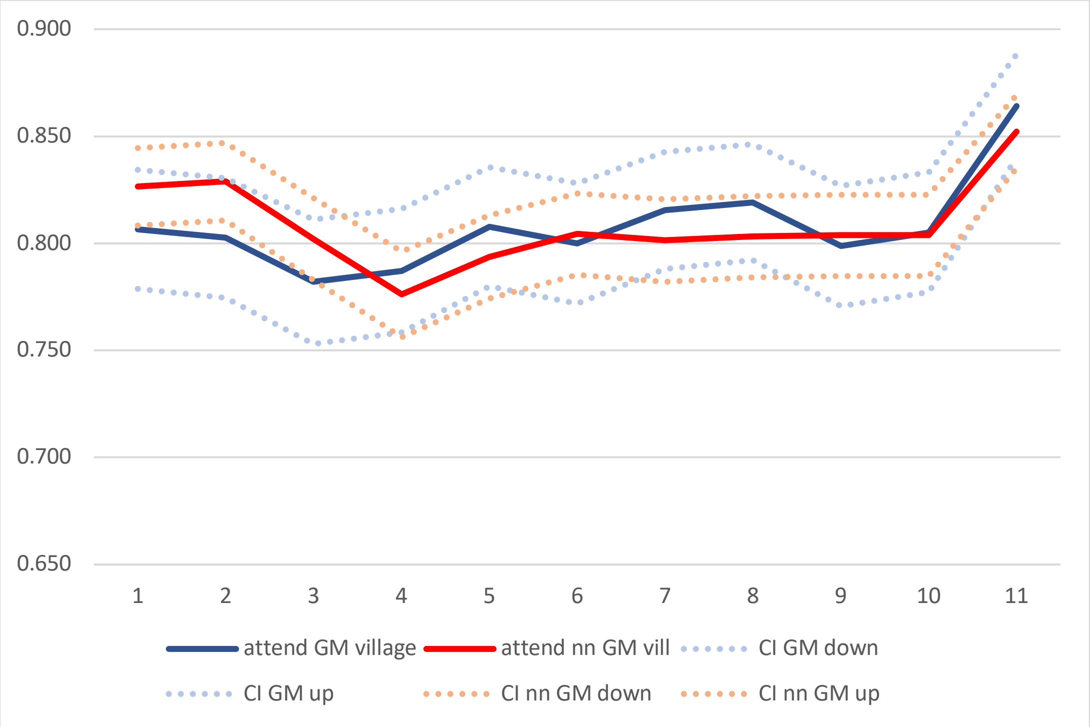

Figure A3a: Retention & acceptability of key messages: main caregivers intensive group trainings (SCESCS-IRC)

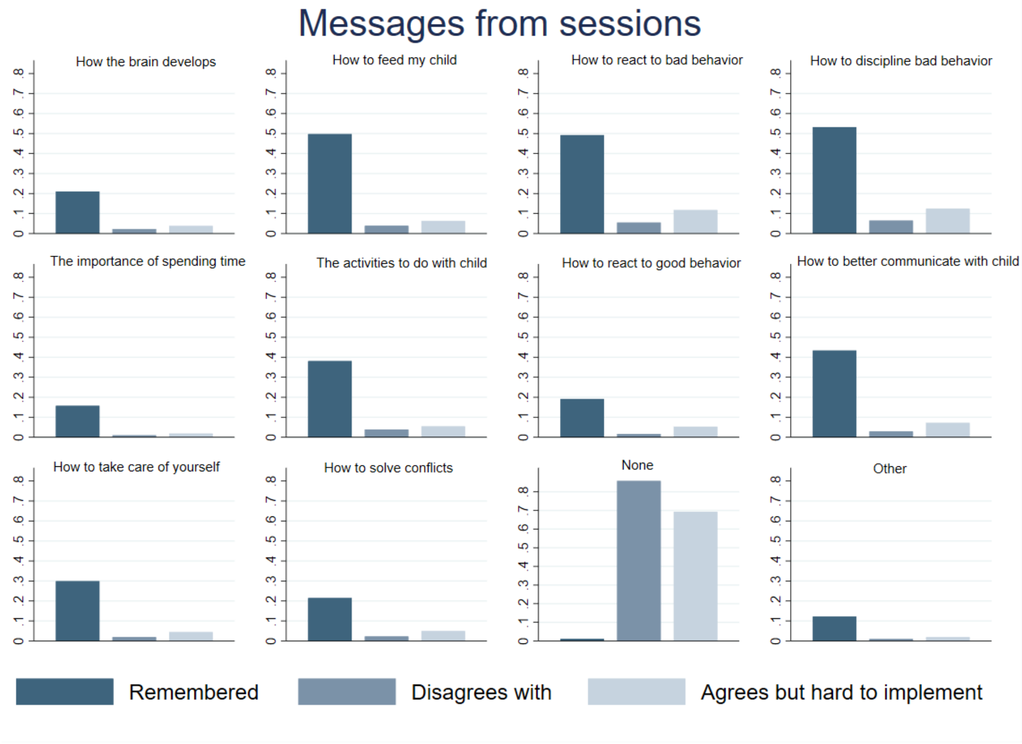

Figure A3b: Retention & acceptability of key messages: grandmothers intensive group trainings (SCESCS-IRC)

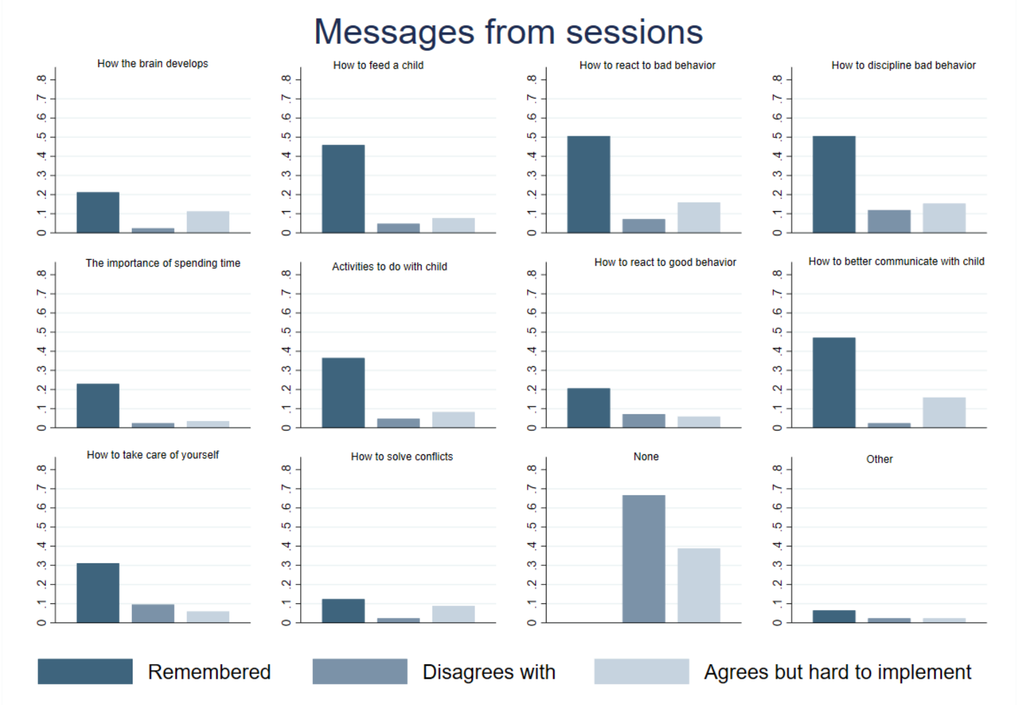

Note: The Figures shows the percentage of participants that mentioned each of the messages when asked about the following questions: (1) Remembered: "Cite three key messages that you remember of that training?" (2) Disagrees with: "What are the advice to which you did not agree?" This question is asked to those that stated NOT to "totally agree" with the messages of the session. (3) Agrees but hard to implement: "What are the advice with which you agreed with but are difficult to put into practice?"
